# Targeting Plastic Exposure in Infertile Couples: A Pilot Intervention Study

**DOI:** 10.3390/toxics14030257

**Published:** 2026-03-16

**Authors:** Jenna Hua, Johanna R. Rochester, Jayne M. Foley, Lindsay B. Hahn, Mia Yan Min, Stacey A. Kenfield, James F. Smith, Shanna H. Swan

**Affiliations:** 1 Million Marker Wellness Inc., Berkeley, CA 94708, USA; jo@millionmarker.com (J.R.R.); jayne@millionmarker.com (J.M.F.); lindsay@millionmarker.com (L.B.H.); 2 Department of Cardiothoracic Surgery, Stanford University, Stanford, CA 94305, USA; yanmin@stanford.edu; 3 Department of Urology, University of San Francisco, San Francisco, CA 94143, USA; stacey.kenfield@ucsf.edu (S.A.K.); james.smith@ucsf.edu (J.F.S.); 4 Fellow Health Inc., San Francisco, CA 94107, USA; 5 Department of Environmental Medicine, Icahn School of Medicine at Mount Sinai, New York, NY 10029, USA

**Keywords:** endocrine-disrupting chemicals (EDCs), bisphenol A (BPA), phthalates, biomonitoring, exposure reduction intervention, environmental health literacy, infertility, semen quality, lifestyle intervention, consumer product substitution

## Abstract

Endocrine-disrupting chemical (EDC) exposure from plastics and everyday products is widespread and linked to infertility. We conducted a 3-month uncontrolled feasibility pilot study among five idiopathically infertile couples to assess whether an intensive lifestyle intervention was associated with within-person changes in urinary EDC biomarkers and exploratory changes in reproductive parameters. The intervention was embedded in a film project (“The Plastic Detox”) and integrated personalized education, product substitutions, at-home urine biomonitoring, sperm testing, and weekly coaching. Urine and semen samples were collected at baseline, 6 weeks, and 12 weeks. Linear mixed-effects models were used to estimate biomarker changes. BPA was designated a priori as the primary biomarker endpoint. Directional reductions were observed in urinary bisphenol A (BPA), mono-n-butyl phthalate (MBP), and monobenzyl phthalate (MBzP) over the intervention period. Within-person reductions in products containing ingredients of concern were associated with lower BPA levels. Descriptive upward trends of semen parameters were observed, with the majority of the subfertile men testing >40 million motile sperm/ejaculate after the intervention. Participants had increased environmental health literacy, were more motivated to reduce exposures, and reported improved wellness endpoints. Four couples achieved pregnancy and live birth during follow-up; given the uncontrolled design and small sample size, these outcomes are presented descriptively. Overall, this pilot study demonstrates feasibility and measurable biomarker change, supporting evaluation in larger, controlled trials.

## 1. Introduction

Endocrine-disrupting chemicals (EDCs), including bisphenols and phthalates, are widely detected in human biomonitoring studies; more than 90% of US adults have measurable concentrations in urine [1,2]. EDCs migrate from food packaging, plastics, personal/household products, and other sources and are linked to adverse reproductive, metabolic, and developmental outcomes and have become a major public health concern [3,4,5,6]. Although infertility is multifactorial, much of it is unexplained; 15–30% of infertility is classified as idiopathic [7,8,9]. However, thousands of studies in humans and animals have linked EDCs to reproductive harm [10,11,12,13,14,15]. For couples attempting conception, EDC exposures may play a role in impairing fertility, gamete quality, and early development [16,17,18,19,20,21], yet practical, evidence-based exposure-reduction interventions have been limited, and few studies have evaluated measurable biomarker changes following structured behavioral programs. 

Given the individualized and resource-intensive nature of exposure-reduction interventions, including repeated biomonitoring and behavioral coaching, we designed this study as a small, uncontrolled feasibility and proof-of-concept pilot. The primary objective was to estimate within-person changes in urinary BPA concentrations over a 12-week intervention period. Secondary objectives included descriptive evaluation of additional EDC biomarkers and semen parameters, while pregnancy and psychosocial outcomes were considered exploratory. This study was not designed to establish causal effects on fertility outcomes but to assess whether measurable biomarker changes could be observed during participation in an intensive lifestyle intervention.

## 2. Methods

For detailed methods, see Supplementary Materials. We embedded an exposure reduction program within a documentary film project (“The Plastic Detox,” released on Netflix in March 2026) focusing on plastics and fertility. Men were recruited from the Fellow Health [22] (direct-to-consumer) semen testing database and social media if they were ≥18 years old, had been attempting pregnancy for 12 months, were current non-smokers, reported no major medical/occupational risk factors for impaired semen quality, lived with a partner, and were not planning fertility treatments or extended travel during the three-month study period. All participants consented to filming and study procedures. This study was designed as a small, uncontrolled feasibility and proof-of-concept pilot to evaluate measurable within-person biomarker changes during participation in an intensive exposure-reduction intervention. The WCG Institutional Review Board approved the protocol (No. 20232187, 22 May 2023).

Six couples were recruited and enrolled between December 2023 and January 2024; one couple dropped out after six weeks, leaving five couples who provided biomarker and lifestyle/wellness data at the 12-week follow-up. Participants were predominantly White, with a mean age of 33, highly educated, and of high income. All couples had been trying to become pregnant for at least 12 months, with one couple unable to conceive for over 10 years. Environmental health literacy (EHL) was generally high, but specific gaps related to plastics were evident. For example, many participants believed storing food in plastic containers was safe and had no opinion about canned foods as a bisphenol source. Financial constraints, lack of actionable guidance, and limited product choices were the most frequently anticipated barriers to exposure reduction. 

## 3. Intervention and Assessments

The intervention was designed to target both behavioral and environmental drivers of exposure. A 12-week design was used based on previous research showing significant reductions in EDC metabolites and improvements in exposure-reduction behavior after 12 weeks [23]. Couples were assessed at three time points: baseline (beginning), 6 weeks (midpoint), and 12 weeks (end). During the assessments, couples completed 24 h “exposure audits” via videoconference, where a Million Marker coach systematically inventoried diet, personal care products, and household products from the prior 24 h. Men collected morning semen samples, and both partners provided two urine samples (first morning void and pre-bedtime). Participants received personalized, actionable urine EDC reports from Million Marker and semen reports from Fellow, followed by a one-on-one coaching session to review results and identify feasible behavior changes. Each household was mailed a vetted box of lower-EDC replacement products (e.g., glass food storage, stainless steel straws, beeswax wraps, fragrance-free products; full list in Supplementary Materials). Weekly personalized coaching and environmental health education sessions for three months reinforced behavior change, problem-solved barriers, and encouraged replacement of higher-EDC products.

## 4. Biomarker Measurement

Urinary metabolites of phthalates, environmental phenols, benzophenones, and BPA (free, glucuronide, and sulfate) were quantified at the UCSF Environmental Biomonitoring Lab using LC–MS/MS in three panels (see Supplementary Materials). BPA was designated a priori as the primary biomarker endpoint based on its relevance to food-contact plastic exposure. Additional metabolites were considered secondary or exploratory outcomes. Creatinine-adjusted concentrations were used when available.

## 5. Statistical Analysis

The primary analytic objective was to estimate within-person changes in urinary BPA concentrations over the 12-week intervention period. Changes in additional biomarkers were evaluated descriptively. We modeled all 18 biomarker concentrations using linear mixed-effects models with time (baseline, 6 weeks, 12 weeks) treated as a categorical fixed effect, with baseline as the reference level. Random intercepts were specified for the individual and couple to account for repeated measures and shared household environment. Random slopes were not included to maintain model parsimony, given the small sample size. Secondary outcomes included descriptive sperm parameters, changes in EHL and readiness to change (i.e., reduce exposures), and wellness outcomes (stress, sleep, BMI, sexual satisfaction).

## 6. Results and Discussion

**Figure 1:**
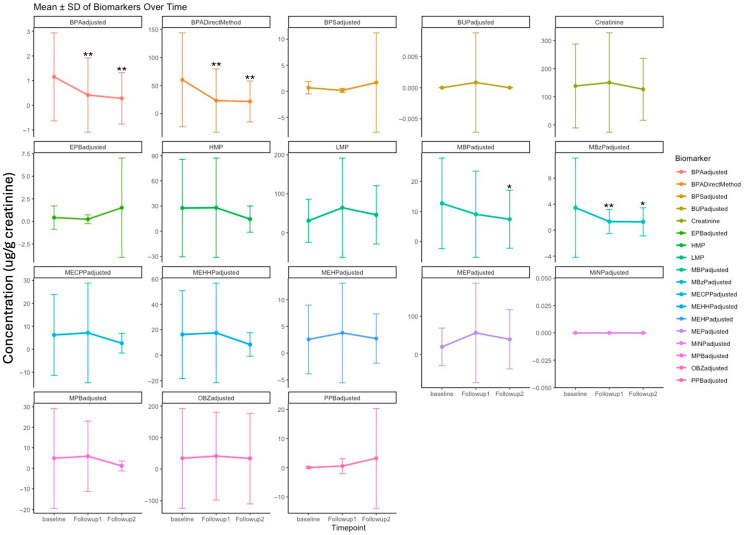
Urine biomarker concentrations (μg/g creatinine) before (baseline, *n* = 12), during (follow-up 1, *n* = 12), and after the intervention (follow-up 2, *n* = 10). * *p* < 0.05 0. and ** *p* < 0.001 in a linear mixed effect model with random effects variables—individual and couple; see Supplementary Materials for abbreviations and methodology.

Five couples provided samples at all three time points, and one couple provided samples at baseline and six weeks. We observed substantial declines in several plastic/plasticizer chemicals over the 12-week intervention (Figure 1). In paired *t*-tests comparing baseline and 12 weeks, urinary BPA concentrations (directly measured and creatinine-standardized) were significantly lower at follow-up (*n* = 10). In mixed-effects models incorporating all three time points, BPA decreased significantly at both 6 (*n* = 12) and 12 weeks (*n* = 10) relative to baseline (*p* ≈ 0.005), with *p*-values presented descriptively. Mono-n-butyl phthalate (MBP) showed a non-significant decrease at six weeks and a statistically significant decrease at 12 weeks, while monobenzyl phthalate (MBzP) decreased significantly at both time points. Monoethyl phthalate (MEP) and MBP trended downward with *p*-values just above 0.05. 

Given the small sample size and exploratory design of this feasibility pilot, statistical tests are interpreted descriptively, and findings beyond the primary endpoint should be considered hypothesis-generating. Adjustment for sociodemographic and health covariates did not materially alter the direction or magnitude of point estimates. 

To link exposure reductions more directly to product use, we constructed an “ingredients of concern” score based on repeated product audits and applied a fixed-effects panel regression with individuals serving as their own controls. Within-person decreases in this score were associated with lower creatinine-standardized urinary BPA (β ≈ −0.008 µg/g per unit decrease; SE 0.0019; within-person R^2^ ≈ 0.26). For context, a 5-unit reduction in the ingredients-of-concern score corresponds to an estimated 0.04 µg/g reduction in BPA, representing approximately 5% of the baseline mean concentration. Similar models suggested that changes in product ingredients also explained a meaningful fraction of the variance in MBP and MBzP, with one-unit changes in ingredients of concern associated with corresponding shifts in these metabolites. Although the directionality for MBP and MBzP varied by metabolite, the overall pattern supports the conclusion that targeted product substitution contributed to lower internal EDC exposures. Because the score was derived independently from product audits and did not incorporate urinary biomarker measurements, these analyses are not circular but instead examine the relationship between documented exposure-related behaviors and internal dose.

**Figure 2:**
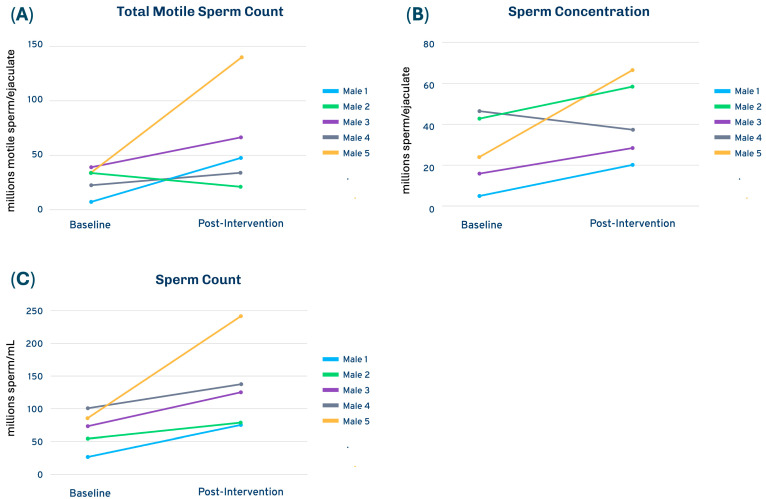
Selected sperm parameters in men with low sperm count (*n* = 5). (**A**) Total motile sperm count (millions of motile sperm/ejaculate; below 40 is subfertile), (**B**) sperm count (millions of sperm/ejaculate), and (**C**) sperm concentration (millions of sperm/mL).

Semen quality was assessed in five men using CLIA-certified analyses at Fellow’s laboratory, including semen volume, sperm concentration, total sperm count, total motile count, motility, and morphology. Although statistical analysis resulted in no significant changes, individual trajectories suggested clinically relevant upward trends in total motile count and concentration (Figure 2). At baseline, all five men had less than 40 million motile sperm/ejaculate, which is considered subfertile for this parameter [24]. After the intervention, three of the men tested higher than 40 million motile sperm/ejaculate (Figure 2A). Importantly, four of the five couples achieved pregnancy and live birth following the 12-week intervention; one couple is expecting their second child. Given the small sample size, short follow-up, lack of abstinence, and inherent variability in semen parameters, these findings should be interpreted cautiously but point to the feasibility and potential value of larger trials powered for reproductive endpoints. 

Beyond biomarker changes, participants experienced both measurable and qualitative benefits from this intervention. At follow-up, EHL [25,26] was significantly improved (*n* = 10). Adjusted analyses accounting for repeated measures and couple-level clustering demonstrated significant increases in EHL knowledge (Δ = 2.0 points; SE 0.57; *p* < 0.01) and EHL attitudes (Δ = 2.2 points; SE 0.88; *p* ≈ 0.02) from baseline to follow-up. Within-person analyses corroborated these findings and revealed the strongest effect for EHL Behavior, which increased by 2.9 points on average (95% CI 2.11–3.69; *p* < 0.001), with all participants showing behavioral improvement. Readiness to Change [23] increased modestly but did not reach statistical significance, likely reflecting ceiling effects in this highly motivated cohort. In contrast, perceived social support, physical activity, self-rated health, and overall product use (i.e., number of products used) remained stable, indicating that observed gains were specific to EHL knowledge, attitudes, and behaviors rather than generalized lifestyle change.

Sexual satisfaction, measured pre/post using the sexual satisfaction index (SSI), was non-significantly increased in women (*n* = 5). At follow-up, five of eight respondents reported increased energy, eight of ten reported improved sleep quality, and all respondents indicated that they planned to maintain lifestyle changes. Several testimonials described improved mood, more regular menstrual cycles, fewer ovulatory symptoms, and even reduced need for stimulant medications. Four of ten participants with post-intervention BMI data had modest weight loss, consistent with emerging evidence linking obesogenic EDCs to weight regulation. Although these secondary outcomes were not the primary focus of this pilot, they suggest that EDC-focused interventions may have broader implications for cardiometabolic and mental health.

This pilot study had important limitations. The sample was small, homogenous, and highly motivated, possibly because participants agreed to be filmed, and several men had low baseline sperm counts. Consequently, replication in larger, more diverse cohorts is necessary to determine the generalizability and robustness of these findings. The intervention was intentionally high-touch, involving intensive coaching, repeated biomonitoring, and provision of free replacement products, which may limit scalability in its current form. Nevertheless, the magnitude and consistency of declines in BPA and several phthalate metabolites over a relatively short period, coupled with the strong within-person association between product ingredients and urinary levels, support a causal role for behavior change in driving exposure reductions. Closely following the study period, four of five couples achieved pregnancy and live birth. While causal inference is not possible in this pilot study, the proportion of pregnancies observed is notable and underscores the need for more rigorous evaluation in larger, controlled cohorts. 

In summary, this proof-of-concept pilot study suggests that plastic-related EDC exposures in infertile couples can be measurably reduced in a timeframe of weeks to months when individuals are provided with personalized biomonitoring feedback, product substitution options, and educational support. Such reductions occurred alongside improvements in selected wellness measures and exploratory reproductive parameters. While these findings are preliminary and not designed to establish causality, they support the feasibility of this intervention model and justify evaluation in larger, controlled, randomized trials. Further studies, including those already underway using the lower-touch, scalable Million Marker program [23,27,28], are needed to evaluate impacts on health and clinical biomarkers, time-to-pregnancy, semen quality, and pregnancy outcomes across more diverse populations. Given the substantial financial burden associated with infertility evaluation and treatment, scalable behavioral interventions that are low risk and relatively low-cost warrant rigorous investigation as potential complements to standard care. If future controlled trials demonstrate clinical benefit, such approaches could meaningfully reduce the economic strain faced by couples trying to conceive. Continued follow-up of this cohort, in which pregnancies and live births in four couples have already been reported, may provide additional insights into longer-term reproductive trajectories after intensive EDC reduction. Finally, the integration of this intervention into a widely disseminated documentary offers a model for combining individualized exposure reduction with public communication campaigns to catalyze broader societal changes in product formulation and consumer behavior.

## Data Availability

De-identified datasets and analysis code are available from the corresponding author upon reasonable request, subject to participant confidentiality and film production agreements.

## Supplementary Materials

The following supporting information can be downloaded at https://www.mdpi.com/article/10.3390/toxics14030257/s1. Supplementary Information: Methods. Table S1: List of clean products provided to participants. Table S2: Metabolites analyzed. See References [22,29,30,31,32,33].
