# Supplementary material for: Targeting Plastic Exposure in Infertile Couples: A Pilot Intervention Study
Source: Toxics. 2026 Mar 16;14(3):257. doi: 10.3390/toxics14030257 (PMC13030407; doi:10.3390/toxics14030257)
Supplement: Supplementary file 1 [file toxics-14-00257-s001.zip › toxics-4167379-supplementary.pdf]

Supplementary Information

Participants. All study materials received approval from the WCG IRB, an independent Institutional Review Board, Protocol #20232187, approved on May 22, 2023. We screened over 100 men and their partners in the Fellow Health database (a direct-to-consumer sperm quality testing company), following the eligibility criteria for the three month intervention: 18 or over; trying to conceive for 12 months or more; no hazardous occupational exposures (i.e. environmental chemicals that can reduce semen quality such as lead or solvents); current non-smoker; no medical conditions that are risk factors for poor semen quality (e.g., obesity, diabetes, liver and kidney diseases, and testicular dysfunction); not expecting a baby; living with a partner, no plans to begin IVF or other fertility treatment during the intervention; no plans to travel during the intervention; willing to be filmed throughout the intervention Participants in the Fellow database were Fellow customers who agreed to be recontacted for research. The final selection of six couples (12 participants) was made by the film team. One of the six couples left the study after six weeks.

Measurements. Upon signing the consent and information release for the film electronically, participants were sent several baseline surveys that assess their demographics, environmental health literacy (EHL), readiness to change (i.e. readiness to implement behaviors to reduce exposures), social support, self-rated health, sleep/stress, physical activity, financial wellbeing, as well as sexual health and satisfaction. These surveys were also administered post-intervention.

Intervention. Participants were sent Million Marker and Fellow test kits. A total of four Million Marker kits (each member of the couple provided AM and PM urine samples) and one Fellow kit were included in the package. Each couple received a package at the start, middle (six weeks), and end (12 weeks) of the three-month intervention (a total of three packages). Participants also received a vetted box of everyday products (household and personal care products) selected by the Million Marker team. A complete list of products provided to the participants can be found in Table S1.

Table S1. List of clean products provided to participants.

	**Products Provided**	**Brand**

**Kitchen**	Beeswax wrap set	LINK
	Silicone bags set	LINK
	Soap (dish, detergent all in one)	LINK
	Glass tupperware set	LINK
	Stainless straws	LINK
	Loofah sponge set	LINK
	Bamboo utensil set	LINK
**Personal Care**	Shampoo bar	LINK
	Deodorant	LINK
	Toothbrush	LINK
	Toothpaste	LINK
	Lip balm	LINK
	Body oil	LINK
	Face oil	LINK
	Shave Soap	LINK
	Sunscreen	LINK
**Bathroom**	Detergent	LINK
	Shower curtain	LINK
**Cleaning/Laundry**	Detergent	LINK

Study participants engaged in the following intervention protocol: 1) Participants were sent Fellow (sperm quality testing, men) and Million Marker (EDC urine test, men and women) test kits. 2) Prior to collecting their urine samples both partners took a comprehensive 24-hour food and product exposure survey administered by a Million Marker coach, who also reviewed the instructions for semen and urine sample collection and returning kits. 3) Men collected morning semen samples and both partners collected two urine samples (first void and before bedtime, in order to capture temporal differences in exposure). Men were instructed to maintain abstinence for 2-7 days prior to the semen collection, as indicated by Fellow's validated protocols [1]. 4) Participants use pre-paid mailers to mail back their samples. 5) Participants received their Fellow semen testing report and reviewed their personalized Million Marker reports and received personalized product recommendations with a Million Marker health coach. 6) Study shipped low-tox products for use during the three-month intervention. 7) Participants received weekly environmental health education and exposure reduction coaching sessions. 8) Participants were re-tested for EDC levels and semen quality at six weeks and three months.

Urine and Sperm Sample Analysis. Urine samples were analyzed at the Environmental Biomonitoring Lab at University of California, San Francisco (UCSF). Upon arrival at the lab, samples were logged, aliquoted and stored in a -80°C freezer, then batched for analysis using liquid chromatography tandem mass spectrometry (LC/MS/MS). Three lab panels using well-established lab methods were run including direct-method bisphenol A (BPA [2]), environmental phenols [3] and phthalates [4]. We included the direct-method BPA panel because it is considered the most sensitive and specific method for measuring levels of BPA in biological samples compared to indirect methods used to measure broader panels of environmental phenols and phthalates [5]. Specific metabolites covered in each of the three panels are listed in Table S2. We assessed the limit of detection (LOD) for each analyte for all three panels by running a series of calibration standards (0.01 – 100 ng/mL), established as the lowest concentration of the analyte that gives a signal/noise (S/N) ratio of 3. We tested all equipment and supplies used in the collection or storage of urine for their potential to contaminate urine specimens. Specifically, we simulated sample collection, extraction and analytical run using synthetic human urine (UTAK Laboratories, Inc.). All processes and equipment were found to be free of contamination (all metabolites < LOD in all field blank testing materials). Analytical reliability and reproducibility were evaluated during method development and validation, including assessment of intra- and inter-assay variability, as described in Gerona et al. 2017 [6].

Sperm samples were processed and analyzed for volume, concentration, motility, total motile count and morphology at Fellow's CLIA-certified lab [1].

Table S2. Metabolites analyzed

Panel Type	Metabolite	Parent Compound	Phthalate Molecular Weight	LOD (ng/mL)
Phthalates	Mononbutyl Phthalate (MBP)	Di-n-butyl phthalate (DBP)	Low	0.05
	Monobenzyl Phthalate (MBzP)	Butyl benzyl phthalate (BBzP)	High	0.1
	Monoethyl Phthalate (MEP)	Diethyl Phthalate (DEP)	Low	0.05
	Mono-2-ethylhexyl phthalate (MEHP)	Di(2-ethylhexyl) phthalate (DEHP)	High	0.05
	Mono-2-ethyl-5-hydroxyhexyl phthalate (MEHHP)	Di(2-ethylhexyl) phthalate (DEHP)	High	0.1
	Mono-isononyl phthalate (MiNP)	Diisononyl phthalate (DINP)	High	10
	Mono-2-ethyl-5-carboxypentyl phthalate (MECPP)	Di(2-ethylhexyl) phthalate (DEHP)	High	0.05
	Mono-hexyl phthalate (MHxP)	Di-n-hexyl phthalate (DnHP)	High	0.1
	Monopentyl phthalate (MPP)	Di(2-ethylhexyl) phthalate (DEHP)	Low	0.1
	Mono-iso-decyl phthalate (MiDP)	Di-iso-decyl phthalate (DiDP)	High	0.05
	Mono-cyclohexyl phthalate (MCHP)	Dicyclohexyl phthalate (DCHP)	High	0.1
Environmental Phenols	Methyl Paraben (MePB)	Methyl Paraben (MePB)	-	0.01
	Ethyl Paraben (EPB)	Ethyl Paraben (EPB)	-	0.01
	Propyl Paraben (PPB)	Propyl Paraben (PPB)	-	0.01

	Butyl Paraben (BUP)	Butyl Paraben (BUP)	-	0.01
	Bisphenol A (BPA)	Bisphenol A (BPA)	-	0.2
	Bisphenol S (BPS)	Bisphenol S (BPS)	-	0.01
	Benzophenone-1 (OBZ)	Oxybenzone	-	0.01
	Benzophenone-3 (OBZ)	Oxybenzone	-	1
	Pentachlorophenol (PCP)	Pentachlorophenol (PCP)	-	0.5
	Triclosan	Triclosan	-	1
Direct BPA	Free Bisphenol-A (BPA)	Bisphenol A (BPA)	-	0.5
	Bisphenol-A (BPA glucuronide)	Bisphenol A (BPA)	-	0.2
	Bisphenol-A (BPA) Sulfate	Bisphenol A (BPA)	-	0.1

Exposure Data. Before each urine collection time point, participants engaged in an exposure audit interview, identifying all personal and household products, foods and drinks, as well as other lifestyle factors that might contribute to EDC exposures in the 24 hours before urine collection. Interviews were conducted over Zoom and lasted between 45 minutes to an hour.

Statistical Analysis.

Urine. Urine metabolites were sampled twice (AM and PM) at three time-points: baseline (N=12), six weeks (follow-up 1, N=12), and 12 weeks (follow-up 2, N=10). Eighteen different biomarkers were assessed. In this analysis, we used creatinine-adjusted values of these biomarkers when available. Changes were assessed using 1) a paired t-test at the baseline and the follow-up 2, and 2) a linear mixed effect model. The random effect reflected the following two variables: individual (to account for repeated measures) and Couple (as a couple shares the same environment, thus their data could be correlated). This analysis included all three time points. For the analysis with confounding variables, we included the following confounders in the mixed effect model: age, sex, education, physical health, personal products (number), household products.

Semen. Five men provided semen samples, at baseline and post-intervention. Analysis included six different semen parameters (total motile count (millions of motile sperm/ejaculate), sample volume (mL), concentration (millions of sperm/mL), motility (%motile), morphology (%non-normal morphology), total count (millions of sperm per/ejaculate)). Analysis was carried out using a linear mixed effect model. The random effect only reflected the different baseline of each individual, with a fixed effect of time. Confounders included age and education. Other possible confounders such as physical health, personal products, and household products are not included due to the lack of variability.

Product Analysis. To examine the relationships between ingredients of concern and the EDC urinary metabolite levels, we employed a fixed-effect panel regression, using repeated measures of creatinine-adjusted urinary BPA (outcome) and ingredients of concern (exposure) for each participant over time. The model featured the "within" estimator: each individual served as their own control. This wiped out all time-invariant differences between participants. The analysis took into account the clustering of the couples as well.

Body mass index (BMI). Changes in BMI from baseline to follow-up were evaluated using a linear mixed-effects model with random intercepts for individuals nested within couples and a fixed effect for time. Variance components were examined to assess between- and within-person variability. A paired t-test comparing baseline and follow-up BMI was also conducted among participants with complete data (N=10).

Survey outcomes: Environmental Health Literacy, Readiness to Change, Physical Activity, Self-Reported Health, Household Product Use. Survey-based outcomes were analyzed using two complementary within-person approaches. For primary environmental health literacy (EHL) outcomes (Knowledge and Attitudes), linear mixed-effects models were used to estimate change from baseline to follow-up, with random intercepts for individuals nested within couples and a fixed effect for time. Models were adjusted for baseline demographic characteristics, including age, sex, race/ethnicity, education, and household income. Social support and sleep outcomes were analyzed using the same modeling framework. In addition, paired t-tests were conducted for survey outcomes with complete baseline–follow-up data (N=10), including EHL Knowledge, Attitudes, Behavior, Readiness to Change, physical activity (MET minutes), self-rated health, and personal and household product use. All survey variables were coded numerically prior to analysis.

Sexual satisfaction. Sexual satisfaction was measured at baseline and final follow-up using the Sex Satisfaction Index (SSI). Because SSI scores are ordinal and sample sizes were small, within-person changes were evaluated separately for men and women using Wilcoxon signed-rank tests. Participants were included if they had both baseline and final measurements, and change scores were calculated to summarize direction and magnitude of change.

All analyses were conducted in R. Two-sided tests were used, with statistical significance defined as $p<0.05$.

References

1. Samplaski, M. K. *et al.* Development and validation of a novel mail-in semen analysis system and the correlation between one hour and delayed semen analysis testing. *Fertil. Steril.* **115**, 922–929 (2021).
2. Gerona, R. R. *et al.* Direct measurement of Bisphenol A (BPA), BPA glucuronide and BPA sulfate in a diverse and low-income population of pregnant women reveals high exposure, with potential implications for previous exposure estimates: a cross-sectional study. *Environ. Health* **15**, 50 (2016).
3. Bloom, M. S. *et al.* Racial differences in the associations between prenatal exposure to environmental phenol mixtures and preterm birth. *Int. J. Hyg. Environ. Health* **271**, 114700 (2026).
4. Begum, T. F. *et al.* A pilot investigation of couple-level phthalates exposure and in vitro fertilization (IVF) outcomes. *Reprod. Toxicol.* **99**, 56–64 (2021).
5. Vandenberg, L. N. *et al.* Urinary, Circulating, and Tissue Biomonitoring Studies Indicate Widespread Exposure to Bisphenol A. *Environ. Health Perspect.* **118**, 1055–1070 (2010).
6. Gerona, R. R. *et al.* Suspect screening of maternal serum to identify new environmental chemical biomonitoring targets using liquid chromatography–quadrupole time-of-flight mass spectrometry. *J. Expo. Sci. Environ. Epidemiol.* **28**, 101–108 (2018).
